# Assessment of Oil Palm Pollinating Weevil (*Elaeidobius kamerunicus*) Population Density in Biparental *dura* × *pisifera* Hybrids on Deep Peat-Soil in Perak State, Malaysia

**DOI:** 10.3390/insects12030221

**Published:** 2021-03-04

**Authors:** Senesie Swaray, Mohd Y. Rafii, Mohd Din Amiruddin, Mohd Firdaus Ismail, Syari Jamian, Momodu Jalloh, Yusuff Oladosu, Mohd Mustakim Mohamad, Marhalil Marjuni, Olalekan Kazeem Kolapo, Samuel Chibuike Chukwu

**Affiliations:** 1 Department of Crop Science, Faculty of Agriculture, Universiti Putra Malaysia (UPM), Serdang 43400 UPM, Selangor, Malaysia; senesieswaray74@gmail.com (S.S.); mohd.firdaus@upm.edu.my (M.F.I.); jalcoke2008@gmail.com (M.J.); 2 Tree Crops Unit, Sierra Leone Agricultural Research Institute (SLARI), Freetown P.M.B 1313, Sierra Leone; 3 Laboratory of Climate-Smart Food Crop Production, Institute of Tropical Agriculture and Food Security, Universiti Putra Malaysia, Serdang 43400 UPM, Selangor, Malaysia; oladosuy@gmail.com (Y.O.); kazeem.olalekan@uniosun.edu.ng (O.K.K.); chukwu.samuel@ebsu.edu.ng (S.C.C.); 4 Advanced Biotechnology and Breeding Centre, Malaysian Palm Oil Board (MPOB), 6 Persiaran Institusi, Bandar Baru Bangi, Kajang 43000, Selangor, Malaysia; mohd.mustakim@mpob.gov.my (M.M.M.); marhalil@mpob.gov.my (M.M.); 5 Department of Plant Protection, Faculty of Agriculture, Universiti Putra Malaysia, Serdang 43400 UPM, Selangor, Malaysia; syari@upm.edu.my

**Keywords:** oil palm, pollination, population density, fruit-set, genetic origins, *Elaeidobius kamerunicus*, hybrids

## Abstract

**Simple Summary:**

*Elaeidobius kamerunicus* is the most efficient pollinator in oil palm plantations. The decline in the oil palm fruit-set and oil yield can be offset with this pollinator weevil. This study measured the population density of *E. kamerunicus* in biparental *dura* × *pisifera* hybrids. The result showed a significant variation in the population density of the weevils among the planting materials. The female population was higher than the male weevil across the hybrids. The highest weevil population was recorded on the third day of anthesis with a decline in its population in subsequent anthesis. The highest mean of *E. kamerunicus* population per spikelet and its population density per month was recorded in January. Hybrid ECPHP550 had the highest mean of *E. kamerunicus* per spikelet and its population density per palm. Hybrid ECPHP550 could be used in palm plantations to maximize the weevil population, especially the male weevil because of its pollen carrying capacity; thus, pollination in palm plantations can be improved in achieving good fruit-set and oil yield.

**Abstract:**

This study was conducted to assess the *Elaeidobius kamerunicus* (EK) population density among the biparental *dura* × *pisifera* hybrids’ palms on deep peat-soil. Twenty-four hybrids derived from 10 genetic sources were used. Variance analysis showed that the EK population density varies between different oil palm hybrids, with a more noticeable variation of a low population mean in the male weevil across the hybrids. The highest weevil population mean/spikelet was attained on the third day of anthesis. The maximum monthly population of EK/spikelet (12.81 ± 0.23) and population density of EK (1846.49 ± 60.69) were recorded in January. Accordingly, 41.67% of the hybrids recorded an EK population density greater than the trial means of 973.68 weevils. Hybrid ECPHP550 had the highest mean of EK/spikelet (10.25 ± 0.11) and the highest population density of EK/palm (1241.39 ± 73.74). The parental mean population was 963.24 weevils and parent Deli-Banting × AVROS recorded the highest EK population density (1173.01). The overall results showed a notable disparity in the EK population among the biparental hybrids. Parental Deli-Banting × AVROS and hybrid ECPHP550 could be more useful to optimize the weevil population for pollination improvements in palm plantations. However, we suggest that volatile production should be included as a desirable trait in oil palm selective breeding.

## 1. Introduction

The increase in global demand for palm oil has promoted the intensification of the African oil palm (*Elaeis guineensis* Jacq) [1,2]. This palm species (*guineensis*) is the world’s leading oil-producing crop, accounting for about 36 percent of the global supply of vegetable oil [3,4]. The cultivation of oil palm has been followed by remarkable success in terms of increases in the gross domestic product (GDP) of oil nations globally [5]. Advances in breeding have led to the development of elite planting materials by the Malaysian Oil Palm Board (MPOB) for the oil palm industry (OPI). MPOB mainly conducted the hybridization for superior hybrids, through an extensive collection of germplasm [6]. According to Chong et al. [7], the consideration towards environmental sustainability has led to increased pressure on the Malaysian OPI to intensify its outputs rather than increasing cultivated areas. With the projected increase in the human population, it is imperative to increase the net exports of vegetable oils and other agricultural products [8]. 

The problems restraining palm oil production should be resolved because of the increased global demand for vegetable oils, and recently as biofuels in several countries [9]. Despite advancements in research and development in oil palm, the yield is still low to match with the growing demands. There has been a rising low fruit set and low oil extraction rate (OER) in Malaysia [10]. Among the causes of this might be attributed to this crop’s narrow genetic base [8]. 

Incomplete pollination coupled with inefficient pollinator operation has resulted in a high number of parthenocarpic fruits [10]. Hence, it is crucial to study pollinator species’ behavior for the better transportation of pollen and the oil palm fruit-set development [11]. The male inflorescences of *Elaeis guineensis* palms are strongly associated with the pollinator *Elaeidobius kamerunicus* Faust (EK), found in the order Coleoptera and family Curculionidae [11]. The utmost important oil palm pollinating insect in Malaysia and other Asian countries is the weevil *E. kamerunicus* [12,13].

Until the late 1970s, assisted pollination or hand-pollination had to be carried out in most oil palm plantations, especially for younger palms, leading to poor yield and fruit production [14]. To overcome inconsistency in pollination and ineffectiveness of locally available oil palm pollinators, the EK was introduced in the early 1980s in Malaysia from Cameroon, to boost fruit-set formation [15]. In 1983, a similar weevil was introduced in Indonesia; as a result, the amount of infertile fruit decreased to approximately 36 percent in commercial plantations [16]. This weevil introduction has vastly improved yield in many palm plantations worldwide [9]. High fresh fruit bunches (FFBs) in oil palm have been recorded due to EK introduction in palm plantations [17]. Kouakou et al. [18] reported that the population abundance of the pollinator weevil is strongly related to the production of the oil palm fruit-set. Male and female inflorescences both emit a distinctive aniseed scent to attract the EK pollinator for pollination [19,20].

The *E. kamerunicus* is dark-brown to black and tiny, with the adult weevil’s average length varying from 1.8 to 4.0 mm [21,22]. Male EK weevils have small tubercle wing cases, a short rostrum, are hairy, and are typically broader (3–4 mm), whereas there is an absence of small tubercles on the wing cases of female, with a longer rostrum, they are less hairy, and smaller, with dimensions of 2–3 mm [23]. EK undergo complete holometabolous, during their period of developmental stages, and their life cycles comprise the egg to larvae (larva stage-I, stage-II, and stage-III), pupa formation, and imago (adulthood) stage, and this holometabolous happens within 10 to 25 days with higher longevity between 15 to 17 days [15,24,25,26].

The weevil is an oil palm-specific host because it does not reproduce on any other species of plants [27]. The soft portion of the oil palm male flowers for egg-laying and pollen production is consumed by the EK pollinator weevil [28]. Kouakou et al. [18] reported that the population abundance of the pollinator weevil is strongly related to the proportion of the oil palm fruit-set.

In Malaysia, the EK efficacy is believed to have decreased [29]. In various plantations, this has resulted in very low fruit-set values of oil palm bunches [30]. Incomplete pollination may cause low bunch development, low fruit, and bunch failure [31]. The pollination effectiveness depends on various factors that regulate insects’ behavior, such as hair quantity and insect size, odor, flower color, weather conditions, and exposure [16]. Prasetyo et al. [29], in their prior findings, attested that the low population density of EK has led to the contemporary decline in fruit-set. Similarly, Frimpong and Adjaloo [17] and Sisye [32] reported that insufficient availability of the pollinating weevil has resulted in the poor fruit-set in oil palm. Given the current yield decline in oil palm, we hypothesized that *Elaeidobius kamerunicus* population density varies between different oil palm hybrids. This study compares EK populations between several biparental *dura* (D) × *pisifera* (P) hybrids’ palms plantation on deep peat soil.

## 2. Materials and Methods

### 2.1. Planting Materials

The planting materials used in this study consisted of 24 D × P hybrids (Table 1) developed through biparental breeding design from cross-pollination of *dura* and *pisifera* parental palms. The parental materials which comprised six female *dura*, namely; Deli Serdang, Tanzania, Angola, Deli Ulu Remis, Deli Johor Labis and Deli Banting, and four *pisiferas*, namely; Cameroon, Algenene-vereniging rubber planters (AVROS), Nigeria and Yangambi were collected from different countries by MPOB for yield improvement and to widen the genetic base of oil palm breeding programs.

### 2.2. Experimental Design and Research Location

**Figure 1:**
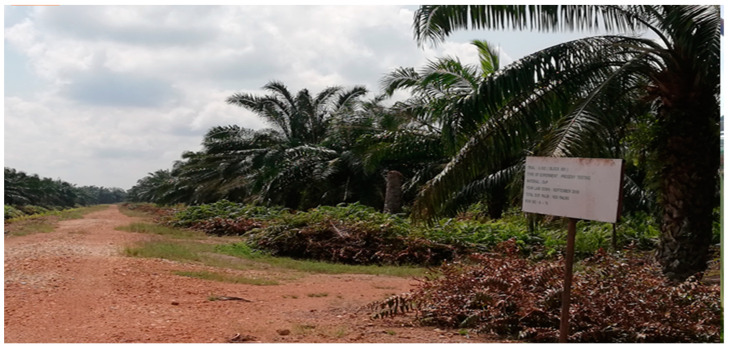
Malaysian Palm Oil Board (MPOB) experimental palms (biparental *dura* × *pisifera* hybrids) at Trial 0.502, Teluk-Intan research station, Malaysia.

The research was conducted at MPOB Teluk-Intan research station (3.49° N, 101.06° E), Bagan-Datuk, Perak State Malaysia (Figure 1). The trial was established in September 2008 in an independent complete randomized design (ICRD) on 12.06 hectares (ha) with 1930 palms. The climate of the location was a hot humid tropic, of an area which fell under the rain forest. The average temperature of the environment was 27 °C, with a minimum of 21 °C, a maximum of 32 °C, and about 85% relative humidity with a total annual average of around 2100 mm of precipitation [33]. The experimental field had a soil pH of 3.4 inches and was graded as a very deep peat-soil of 33.6% carbon content with homogenous soil setting for this study, and the location of the trial plot was flat, with uniform soil conditions, and a moderate distribution of rainfall [34]. In the current study, systematic random sampling was employed for the palm selection process per hybrid, in which three palms/hybrid out of 16 or 14 palms for each hybrid per replication, making a total of 12 palms/hybrid, was selected (3 palms × 4 replications = 12 palms/hybrid). A total of 288 sample palms were selected for this study (12 sample palms/hybrid × 24 hybrids = 288 sample palms or 3 × 4 × 24 = 288 sample palms).

### 2.3. Procedure and Data Collection on the Elaeidobius kamerunicus Population Density in Oil Palm Hybrids

Assessments of *E. kamerunicus* population density on anthesis days, and sex determination of the pollinator among the hybrids were carried out following the methods of Basri and Norman [30] and Chiu et al. [35]. The plant itself is dioecious, in which the inflorescences of both males and females appear separately. The male inflorescence comprises a 40 cm stout peduncle carrying about 100 to 300 spikelets, each measuring 10–30 cm in length, and normally, each of the spikelets contains 400 to 1500 male flowers [36]. There is a shorter and stouter stalk in the female inflorescence, which carries 150 spikelets that each contain 30 flowers [36]. The categorization of the male inflorescences which were approximately 25% opened and functional were labeled as day one of anthesis. On day two, the opening of the flowers increased approximately by 50%, and by 100% on day three. Data on the oil palm pollinator insect (*E. kamerunicus*) population density comprised male (M) and female (F) EKs, number of spikelets per male inflorescence at anthesis (NS/MI), number of male inflorescences at anthesis (NMI/A), number of female inflorescences at anthesis (NFI/A), and the length of spikelets (SPL cm) were collected on a monthly bases for twelve months (February 2019 to January 2020).

**Figure 2:**
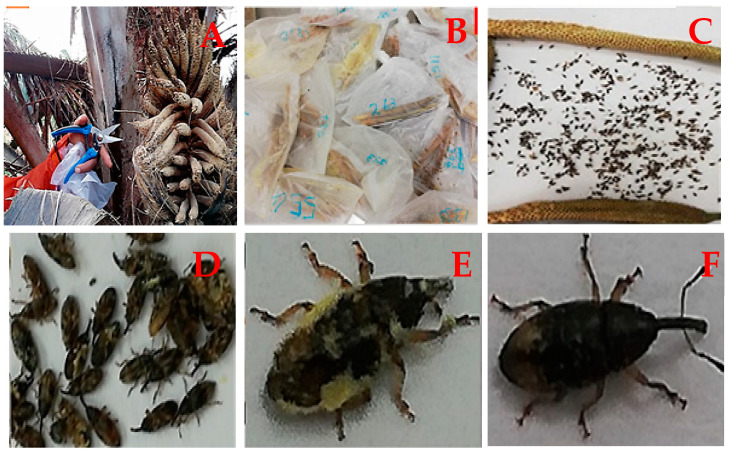
Collected samples of *E. kamerunicus* and determination of male and female: (**A**) harvesting of anthesising male inflorescence spikelets; (**B**) collected samples of *E. kamerunicus*; (**C**) spikelets and pollinator weevils; (**D**) assorted male and female *E. kamerunicus*; (**E**) male lateral view of *E. kamerunicus*; (**F**) female lateral view of *E. Kamerunicus.*

During anthesis stages, each selected hybrid palm was visited from the first to the sixth day of anthesis, and a total of 18 spikelets were harvested. In the first three days, a total of nine spikelets were collected for determination of the EK population density. Equal time interval sampling collections of each hybrid were observed within the hours of 07:00 a.m. to 10:00 a.m. At each visit, three spikelets (base, medium and apical portions) were selected and harvested at different angles using a pair of secateurs (Figure 2). The harvested spikelets were stored in labeled plastic containers according to palm number. The weevils were sorted into male and female and their population densities were determined (Figure 2). A magnifying glass was used to differentiate between the male and female EK using the three distinct characteristics (proboscis, thorax, and body-hair) (Figure 2). The total EK pollinator density was also determined following Basri and Norman [30]. The mean of the pollinator insects per spikelet was determined based on the anthesis phases. On the last day of anthesis, the male inflorescences were harvested from the main palm and the number of spikelets was determined for further calculations.

### 2.4. Data Analysis

The mean data were subjected to analysis of variance using a general linear model (PROC GLM) of the Statistical Analysis System (SAS) version 9.4 (SAS Institute, Cary, NC, USA). The variance component (VARCOMP) (variance in the number of weevils between palm genotypes (σ^2^_g_), error variance (σ^2^_e_), and phenotypic variance (σ^2^_ph_)) estimate was also carried out using the restricted maximum likelihood (REML) procedure. For multiple mean comparisons, a 5% probability level following Tukey’s studentized range (HSD), and descriptive statistics (mean and standard error) were calculated. Pearson’s correlation coefficient (r) analysis was carried out to measure the relationship of the EK population density with days of anthesis, EK population per spikelet, number of spikelets per male inflorescence, and spikelet length.

## 3. Results

### 3.1. ANOVA and Variance Component of the Elaeidobius kamerunicus Population on Each Day of Anthesis

The analysis of variance (ANOVA) in Table 2 showed highly significant differences at *p* ˂ 0.01 for all the parameters at each anthesis days (1–6) for male weevils (MD2 (male day-two), MD3 (male day-three) and MD5 (male day-five)), female weevils (FD1 (female day-one), FD2 (female day-two), FD3 (female day-three), FD4 (female day-four), FD5 (female day-five) and FD6 (female day-six)), and both male and female weevils (EKD1 (EK day-one), EKD2 (EK day-two), EKD3 (EK day-three), EKD4 (EK day-four), EKD5 (EK day-five) and EKD6 (EK day-six)), except for male day-one (MD1), male day-four (MD4) and male day-six (MD6), which remained to be insignificant. There was no significant difference observed among the replications. The analysis revealed that the variance in the number of male weevils between palm genotypes (σ^2^_g_) from day-one to day-six for the male population ranged from 7.50% to 53.33%. The highest σ^2^_g_ for the male population was MD3 of 53.33% followed by MD2 of 50.00%, and the lowest σ^2^_g_ was noticed in MD6 at 7.50%. However, the male population had an error variance (σ^2^_e_) ranging from 7.43% to 92.50% from day-one to day-six of anthesis phases. The MD6 recorded the highest error variance (σ^2^_e_), of 92.50%.

Additionally, the variance in the number of female weevils between palm genotypes (σ^2^_g_) from anthesis day-one to anthesis day-six varied from 35.29% to 63.64% (Table 1). The least σ^2^_g_ was noticed in female day-five at 35.29%, while the highest σ^2^_g_ for the female population was noticed at FD3: 63.64%. Similarly, the error variance (σ^2^_e_) for female EK varied from 36.66% to 64.71%. The FD3 had the lowest σ^2^_e_ and FD5 had the highest, followed by FD6 (Table 2). The EK population variance component by day is presented in Table 2. The range of 37.04% to 65.48% accounted for σ^2^_g_, of which EKD3 had the highest σ^2^_g_ (65.48%), with the least variance in the number of weevils between palm genotypes of 37.04% for EKD5. Likewise, the lowest σ^2^_e_ of 34.52% was noticed in EKD3. The σ^2^_e_ values for EKD5, EKD4 and EKD6 of 62.96%, 56.19%, and 52.50%, respectively, were the highest σ^2^_e_.

#### 3.1.1. Daily Population Mean ± Standard Error of Male *Elaeidobius kamerunicus* per Male Inflorescence Spikelet at Each Day of Anthesis

**Figure 3:**
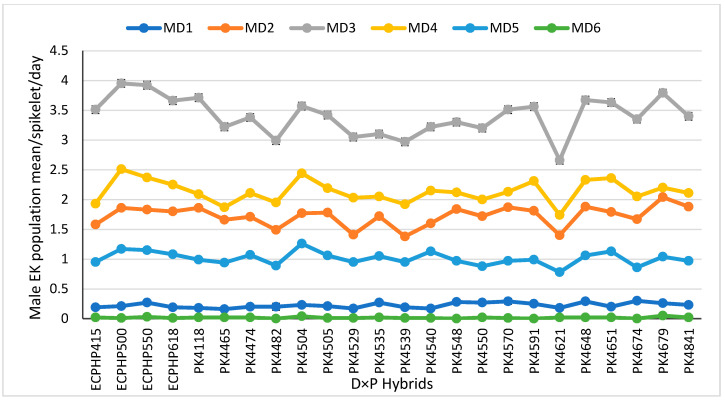
Population means of male *Elaeidobius kamerunicus* per spikelet on each stage of anthesis in biparental hybrids. Mean ± standard error are presented in the figure as each data point: MD1 (male day-one), MD2 (male day-two), MD3 (male day-three), MD4 (male day-four), MD5 (male day-five), MD6 (male day-six); ECPHP, *Elaeis guineensis* crossing program Hulu Paka; PK, Porim Kluang; Tukey’s studentized range (HSD) at *p* ˂ 0.05.

The average population of the male pollinator weevil in individual hybrids on each day of anthesis is presented in Figure 3. Based on the statistical analysis, Tukey’s test (*p* < 0.05) showed that the male EK population mean among the hybrids on each day of anthesis exhibited significant variations. However, there were no significant differences for the male population amid the hybrids on the first and sixth days of anthesis per spikelet, with trial means of 0.22 ± 0.01 and 0.02 ± 0.00, respectively. The male population mean of *E. kamerunicus* per spikelet increased from day one to day three, and the population declined from day four to the sixth day in the same trend for all the hybrids, while no adult EK was found on the spikelets on the sixth day (Figure 3). Tukey’s test showed a significant difference among the oil palm biparental D × P hybrids for the weevil population on the second day of anthesis. Based on the mean separation analysis, 13 out of 24 hybrids with a trial mean of 1.73 ± 0.02 male weevil per spikelet recorded individual mean populations of EK above the trial mean. Hybrid PK4679 had a maximum EK population per spikelet of 2.04 ± 0.09, and 1.38 ± 0.07 was the lowest recorded by PK4539. On the third day of anthesis, a trial mean of 3.42 ± 0.04 was recorded for MD3, with a significant difference amid the hybrids. However, 12 or 50% of the families recorded a weevil population above the trial mean for MD3. Hybrids ECPHP500 and ECPHP550 had the highest weevil population per spikelet with no significant difference between them with mean values of 3.95 ± 0.16 and 3.92 ± 0.12, respectively, whereas the lowest of 2.66 ± 0.63 was noticed in PK4621.

Moreover, on the fourth day of anthesis, a sharp decline in the male EK population mean was significant among the hybrids. The MD4 weevil population trial mean was 2.14 ± 0.03; the lowest and highest means for MD4 were recorded by hybrids PK4621 and ECPHP550, respectively (Figure 3). On the fifth day of anthesis, the male population declined with a significant difference between the hybrids. The trial mean for MD5 was noticed as 1.02 ± 0.02. Nevertheless, PK4504 had the highest male weevil population per spikelet (1.26 ± 0.06), and hybrid PK4621 recorded the fewest weevils per spikelet. As the male weevil population mean continued to decline, there was no significant difference among the hybrids for MD6 found, because no adult male weevil was found on the spikelets (Figure 3).

#### 3.1.2. Daily Population Mean ± Standard Error of Female *E. kamerunicus* per Spikelet

**Figure 4:**
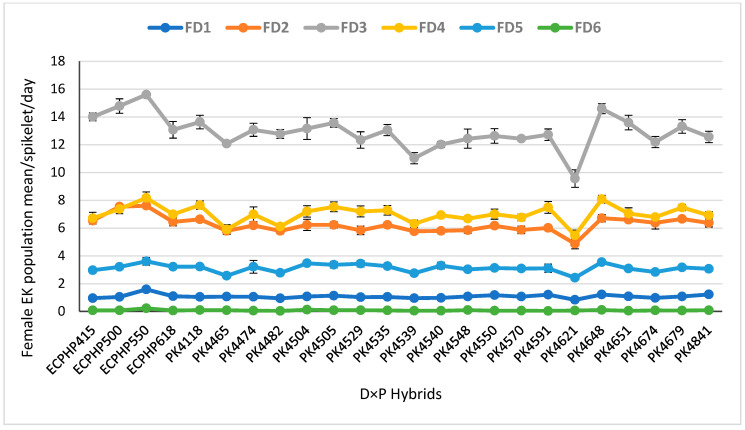
Female *Elaeidobius kamerunicus* population mean per spikelet on each anthesis phase in biparental hybrids. Mean ± standard errors are presented in the figure as each data point: FD1 (female day-one), FD2 (female day-two), FD3 (female day-three), FD4 (female day-four), FD5 (female day-five), FD6 (female day-six); ECPHP, *Elaeis guineensis* crossing program Hulu Paka; PK, Porim Kluang; Tukey’s studentized range (HSD) at *p* ˂ 0.05.

Tukey’s test showed that the days of anthesis manifested a highly significant effect among the hybrids on the female EK population mean per spikelet. The female pollinating weevil population increased from day one to day three and declined on the subsequent days of anthesis (Figure 4). The trend at which the female pollinating weevil increased (1–3 days) and declined (4–6 days) occurred across the biparental D × P hybrids. The FD1 population had a trial mean of 1.08 ± 0.02, and ECPHP550 recorded the highest female weevil population mean of 1.58 ± 0.06 on the first day of anthesis, while hybrid PK4621 recorded the lowest of 0.83 ± 0.04. On the second day of anthesis, the trial mean for the FD2 was 6.27 ± 0.07, and 50% of the hybrids had a mean value greater than the trial mean. Hybrid ECPHP550 had a maximum female population weevil of 7.60 ± 0.16 per spikelet and PK4621 had the lowest female EK population of 4.86 ± 0.36 per spikelet. Additionally, 50% of the hybrids had a female weevil population greater than the general mean (12.94 ± 0.16) for FD3. Hybrids ECPHP550 and PK4621 continued to record the maximum and minimum population mean of EK, respectively (Figure 4).

Nevertheless, FD4 trial mean was recorded as 7.02 ± 0.09, when the female population started to decline. The lowest and highest means for FD4 were recorded by hybrids PK4621 and ECPHP550, respectively (Figure 4). On the fifth day of anthesis, the female EK population continued to decline, but with a significant difference among the hybrids. An experimental mean for FD5 was noticed as 3.13 ± 0.04. However, the analysis showed that hybrids PK4648 (3.54 ± 0.14) and ECPHP550 (3.60 ± 0.28) recorded the highest female EK on the fifth day of anthesis, and hybrid PK4621 (2.42 ± 0.06) recorded the fewest weevils per spikelet. Eventually, a statistically significant difference occurred among the hybrids on FD6 with a single hybrid being significant, while no significant difference was noticed among the biparental hybrids for the population mean of the female pollinator per spikelet.

#### 3.1.3. Daily Population Means of *E. kamerunicus* per Spikelet of Each Anthesis Days (1–6)

**Figure 5:**
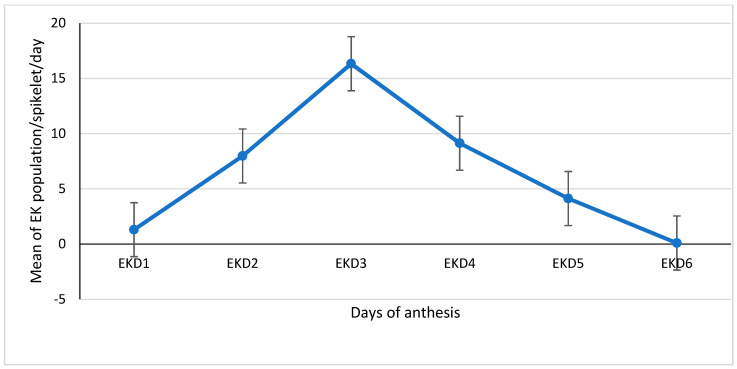
Mean of all hybrids of *Elaeidobius kamerunicus* (EK) population that emerged on male inflorescence per spikelet on each anthesis day (1–6). Means and the standard error are presented in the figure as each data point: EKD1 (EK day-one), EKD2 (EK day-two), EKD3 (EK day-three), EKD4 (EK day-four), EKD5 (EK day-five), and EKD6 (EK day-six).

The population means of *E. kamerunius* in each hybrid for each day of anthesis on anthesising male inflorescence spikelets were categorized according to anthesis days, and the mean of each day is presented in Figure 5. In the oil palm pollination process, the emphasis is on male inflorescence and the pollinator weevil. The illustration in Figure 5 exhibits the population density on each anthesising day (1–6). The EK population mean on the first day (EKD1) and second day (EKD2) of anthesis was 1.3 and 7.97 weevils per spikelet, respectively. On the third day (EKD3), the population mean further increased to 16.32 weevils per spikelet.

However, a decline in the weevil population was noticed on the fourth day (EKD4) of anthesis, with a mean value of 9.13 weevils per spikelet. The population mean of the weevil per spikelet continued to decrease on the fifth day (EKD5). The EK population further declined on the sixth day (EKD6) and had a mean of 0.09 weevils per spikelet (Figure 5).

### 3.2. Monthly Population Mean of E. kamerunicus per Spikelet and its Population Density per Hybrid

A total of 12 months of data collection on *E. kamerunicus* population density in D × P hybrids were carried out from February 2019 and ended in January 2020. The monthly means of *E. kamerunicus* per spikelet, and the monthly population means of the weevils are presented in Table 3. The analysis of variance showed that the replications had no significant difference for a mean number of E. kamerunicus per spikelet (MEK/S) and population density of *E. kamerunicus* (PD/EK), but for the two parameters, a significant difference was noticed among the 12 months and the hybrids. Similarly, a significant interaction occurred between months and hybrids for MEK/S, and there was a statistically significant difference for PD/EK.

Moreover, there was a significant difference in both of the measured parameters amongst the hybrids for the EK population. The *E. kamerunicus* population sample for August and September 2019 for each of the two parameters recorded the smallest MEK/S and PD/EK, with no significant difference between the two months (August 3.96 ± 0.12 and 360.63 ± 14.24 and September 4.36 ± 0.17 and 359.57 ± 16.71, respectively) (Table 3).

Furthermore, the analysis showed that a significant variation for the two parameters (MEK/S and PD/EK) among the hybrids occurred (Table 3). The MEK/S had an experimental mean of 8.55 ± 0.10, and the trial mean for PD/EK was 973.68 ± 18.31. The overall mean performance was 47.67%, or 10 out of 24 hybrids above the trial mean. The mean comparison analysis showed that hybrid PK4621 recorded the lowest EK population for both traits (MEK/S 6.49 ± 0.33 and PD/EK 629.90 ± 44.09). In this study, hybrid ECPHP550 recorded the maximum MEK/S (10.25 ± 0.11) followed by ECPHP500 (9.80 ± 0.11). The highest PD/EK was recorded by ECPHP550 (1241.39 ± 73.74) followed by ECPHP500 (1226.82 ± 61.21).

### 3.3. Mean Number of Spikelets per Male Inflorescence, Spikelet Length, and E. kamerunicus Population Mean among Parental Origins

**Figure 6:**
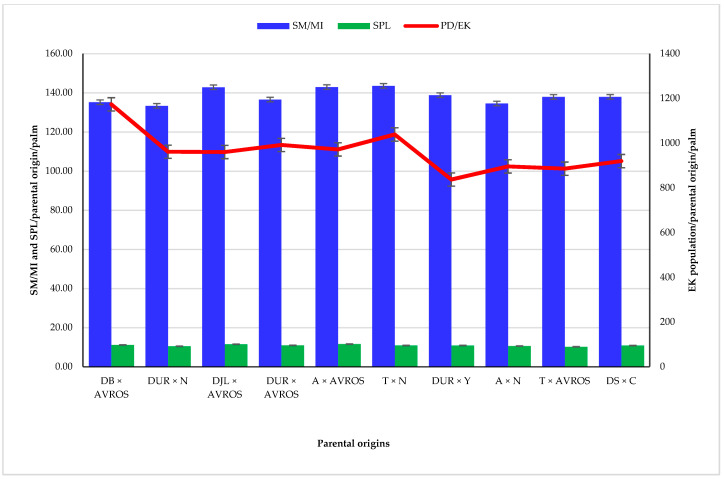
Spikelet means per male inflorescence and population means of *Elaeidobius kamerunicus* on male inflorescence among the *dura* and *pisifera* genetic origins: SM/MI, number of spikelets per male inflorescence; PD/EK population force of *Elaeidobius kamerunicus*; AVROS, Algemene Vereniging Rubber planters; DB× AVROS, Deli Banting × AVROS; DUR×N, Deli Ulu Remis × Nigeria; DJL× AVROS, Deli Johor Labis; DUR × AVROS, Deli Ulu Remis × AVROS; A× AVROS, Angola × AVROS; T × N, Tanzania × Nigeria; DUR × Y, Deli Ulu Remis × Yangambi; A × N, Angola × Nigeria; T × A, Tanzania × AVROS; DS × C, Deli Serdang × Cameroon.

A total of 24 D × P hybrids were grouped according to their parental origins for mean spikelets per male inflorescence (SM/MI), spikelet length (SPL), and population means of *E. kamerunicus* (Figure 6). The parental means of the three traits were SM/MI (138.41), SPL (10.98), and PD/EK (963.24). Genetic origin Deli Ulu Remis × Nigeria (DU × N) constituted the least abundant SM/MI (133.39), whereas Tanzania × Nigeria (T × N) produced the highest SM/MI (143.61) followed by Angola × Algemene Vereniging Rubber planters (A × AVROS) with 143.00 SM/MI, and Deli Johor Labis × AVROS (DJL × AVROS) which had an SM/MI of 142.88.

Angola × AVROS (A× AVROS) produced the longest SPL at 11.68 cm followed by Deli Johor Labis × AVROS (DJL × AVROS) at 11.57 cm, and the shortest SLP was observed in Deli Serdang × Cameroon (DS × C) at 10.9 cm. The maximum PD/EK abundance was recorded by genetic origin Deli Batang × AVROS (DB × AVROS) at 1173.01, followed by T × N at 1038.33, and the minimum of 836.85 PD/EK was noticed in parental Deli Ulu Remis × Yangambi (DUR × Y).

### 3.4. Phenotypic Correlation Analysis

The phenotypic relationship that occurs between two parameters within individuals, which could be either negative or positive, is a correlation. The characters’ phenotypic correlations were measured using Pearson’s correlation at *p* ≤ 0.05 significance levels and *p* ≤ 0.01 high significance levels. Determination of the correlation coefficient (*r*) was estimated thus: perfect relationship (0.90 < *r* =1), strong (0.75 ≤ *r* ≤ 0.90), moderate (0.5 ≤ *r* ≤ 0.75) and weak correlation (*r* ˂ 0.50). Where *r* = correlation value, df = degree of freedom for both replications and hybrids, and *p* = probability value. Spikelet length (SPL) was correlated with the EK population on anthesis days (EKD1, EKD2, EKD3, EKD4, EKD5, and EKD6). The mean of *E. kamerunicus* per spikelet (MEK/S) was correlated with SPL, EKD1, EKD2, EKD3, EKD4, EKD5, and EKD6. Moreover, PD/EK was correlated with SPL, MEK/S, and spikelets mean per male inflorescence (SM/MI). The analysis showed that SPL had a high weak positive correlation with the weevil population density on EKD1 (*r* = 0.124; df = 3, 23; *p* = 0.0001), EKD2 (*r* = 0.198, df = 3, 23; *p* = 0.0001), EKD3 (*r* = 0.202; df = 3. 23; *p* = 0.0001), EKD4 (*r* = 0.204; df = 3, 23; *p* = 0.0001), EKD5 (*r* = 0.214; df = 3, 23; *p* = 0.0001) and EKD6 (*r* = 0.116; df = 3, 23; *p* = 0.0002).

However, MEK/S had a perfect correlation with the weevil population density on EKD2 (*r* = 0.948, df = 3, 23; *p* = 0.0001), EKD3 (*r* = 0.985; df = 3. 23; *p* =0. 0001), but a positive moderate relationship was observed between MEK/S and the pollinator weevil population on EKD1 (*r* = 0.704; df = 3, 23; *p* = 0.0001), EKD4 (*r* = 0.744; df = 3, 23; *p* = 0.0001) and EKD5 (*r* = 0.688; df = 3, 23; *p* = 0.0001), while a weak positive relationship was noticed between MEK/S with the weevil population on EKD6 (*r* = 0.158; df = 3, 23; *p* = 0.0001) and SPL (*r* = 0.205; df = 3, 23; *p* = 0.0001). Similarly, a weak positive correlation was observed between PD/EK and SPL (*r* = 0.190; df = 3, 23; *p* = 0.0001). However, PD/EK had a strong positive relationship with MEK/S (*r* = 0.795; df = 3, 23; *p* = 0.0001), and it was also observed that PD/EK had a moderate correlation with SM/MI (*r* = 0.722; df = 3, 23; *p* = 0.0001).

## 4. Discussion

One of the most substantial criteria used to apportion resources for the most promising genotypes to improve oil palm productivity through breeding programs is selecting suitable parents [37]. Among oleaginous crops, oil palm is the most oil yielding crop and produces an average of four to six tonnes of palm oil per hectare. With the growing global population and palm oil consumption, demand for edible oil by 2050 is expected to reach 240 million tonnes [38]. In the pollination of oil palm plants, *E. kamerunicus* has a significant role [22]. The current study showed significant variation in *E. kamerunicus* population density among the hybrids. The result was in agreement with Abd-Latip et al. [39], who reported significant differences in EK population among the D × P hybrid planting materials.

The variance component showed that variance in the number of weevils between palm genotypes has a greater effect on the weevil population, whereas the environmental effect was more noticeable on the fourth to the last phase of anthesis. The influence of variance in the number of weevils between palm genotypes on the *E. kamerunicus* population could be due to the scent/aroma produced by the male inflorescence at the time of anthesis, based on personal field observation and what was previously reported in the analyses of the volatile organic compound in inflorescences of *Elaeis guineenses* by Muhamad-Fahmi et al. [19]. Estragole is the prime volatile compound found in oil palms’ inflorescences as the foremost attractant compound in the male spikelet of the Africa oil palm that attracts the oil palm pollinator *E. kamerunicus* [19]. Similarly, Nasir et al. [22] reported that pollination in oil palm occurs because the scent of the palm flowers, especially male flowers with a stronger scent/aroma than the female flowers, is attractive to the EK weevil. The pollen will adhere to its body while perched on the male flowers, and then while perched on the blooming (receptive) female flower, the weevil releases the pollen and the female flowers are pollinated [22,40]. An increase in EK population density started at the onset of anthesis to the third day and started declining on the fourth day. This follows the findings of Yue et al. [41], who reported that the weevil population increased from day-one to day-three and declined from day-four to the sixth day of anthesis. EK population on each day of anthesis among the hybrids strongly depends on the scent/aroma produced by the male inflorescences due to the genetic makeup of the palms. This result was supported by Muhammad-Fahmi et al. [19], who determined the aroma produced by male inflorescence to be estragole/P-methoxyally benzene. Therefore, D × P hybrids with a stronger estragole/P-methoxyally benzene (aroma) had an increase in the population density of the weevils. This was supported by Fatihah et al. [42], who reported that pollen coupled with aroma produced by flowers generally fascinated the insect population.

A significant difference in the male weevil population among the hybrids was observed with an increase in the population means from the first day of anthesis to the third day, which declined on the fourth to the fifth day. No adult male weevil was found on the male inflorescence spikelet on the sixth day of all the hybrids. This may be due to the reduction in the scent/aroma produced and a decrease in pollen availability. It was noticed that the days of anthesis manifested a highly significant effect among the hybrids on female EK population force per spikelet. The female pollinating weevil means increased in the same trend as the male but with a higher population mean. The trend in which the female pollinating weevil increased (1–3 days) and declined (4–6 days) occurred across the D × P hybrids. The results showed that the female weevil population mean was greater than the male weevil population on anthesising male spikelets. This follows the previous findings by Yue et al. [41], who reported that the number of female *E. kamerunicus* on anthesising male spikelets was continuously greater than the number of male weevils. Besides, male weevil’s lower population may be attributed to the hatching of females from the mother weevil’s unfertilized eggs, or perhaps the likelihoods for mating are occasional; this needs further study.

Conspicuously, due to a smaller number of *E. kamerunicus*, this natural pollinator’s pollen carrying capacity also decreased, which eventually contributes to pollination failure [9]. Similarly, poor pollen quality of some genotypes and a decrease in female flower germination may also be other causes of variation in the weevil population among the hybrids. However, agricultural pollination experiments have resulted in a possible substantial increase in crop production levels. The germination of pollen is a function of hydration-speed; the speed at which the tip of the pollen tube could be ready to form a tube that develops via the wall of the pollen [43]. Pollen sprouting and viability analysis are valuable tools, because pollen germination rates are proportional to the number of seeds and the production of fruits [44]. Studies by Moura et al. [45] have revealed that low pollen viability decreases fruit production in hybrids of *Elaeis guineensis.* Moura et al. [45] further reported that between 2:00 and 4:00 p.m., *E. kamerunicus* showed no pollinating activity in female flowers. Unsatisfactory pollination could result in a poor fruit-set and lead to a fruit bunch failure and a loss of oil yield [46]. Harun et al. [46] further reported that the high parthenocarpic fruit ratio in the inner bunch suggested that the flowers’ fertilization was not excessively effective due to the thick packing of flowers blocking the pollen’s entry.

However, the weevil’s abundance appeared to be greatly influenced by the production of the male flowers. The earlier study carried out by Luqman et al. [47] reported that due to the occurrence of more male flowers, where *E. kamerunicus* animate, breed, and feed (they are extremely reliant on male flowers), the abundance of the weevil in oil palm plantation will be high. The release of the aroma by the male flowers depends on the florets opening at 25%, 50%, and 100% on the first three days of anthesis, which initiated an increase in the number of the weevils per spikelet [41]. This indicated that fewer EKs were found on the anthesising male spikelets on the first day of anthesis, with the same trend across the hybrids. A further increase in the number of weevils on anthesising male spikelets on the second day was observed. The EK continued to increase in number when the florets had completely opened on the third day of anthesis. The number of EK likely declined on the fourth and fifth days and the sixth day; no adult EK was found on the anthesising male spikelets. These findings were certainly in line with those of Yue et al. [41]. During the 12 months of sampling, the first three days of population mean per spikelet was significantly higher in January, while the lowest MEK/S did not differ significantly in August and September. It was noted that hybrid PK4621 had the fewest, whereas ECPHP550 recorded the highest population, followed by hybrid ECPHP500.

According to Basri and Norman [30], 15 to 30 normal *E. kamerunicus* per spikelet are required for good pollination in oil palm. Rizuan et al. [48] reported that 20 weevils per spikelet were essential. Yue et al. [41] reported that the highest mean number of weevils on the anthesising male spikelet was 33 per spikelet. These varying reports could be due to soil type, the genetic of the planted sampled materials, and the palm age. Based on the present study’s findings, the density of the EK population per anthesising male spikelet was lower than those obtained by previous researchers. The variation in EK population among the hybrids may be as a result of natural enemies, which included, but were not limited to, rodents, birds, and spiders as the most compelling evidence. Earlier studies showed that the larvae of this important and efficient oil palm pollinator weevil had been used as feed by predators such *Pycnonotus goiavier* bird species, ants, and rats [41,49]. An increase in the *Rattus tiomanicus* population caused extreme damage to oil palm inflorescences [50]. Indications of damage caused by these natural enemies in oil palm plantations were frequently observed at Trial 0.502.

Significant differences in EK population density existed among the D × P hybrids and interaction between sampling months and hybrids. This result indicated that months of data collection also led to variation in the EK population amid the D × P hybrids. Fatihah et al. [42] mentioned that the amount of precipitation was associated positively with the oil palm weevil population. The lowest population density was recorded in August and September. However, the highest PD/EK was recorded in January. Water stress could affect the plants’ growth and yield, ultimately affecting the insect community; hence, rainfall might influence the abundance of insects [2]. Very low rainfall remained unsatisfactory for the weevil activity in Riau-Sumatra, and due to the pollen compactness on the body of the weevil, the wet-season could result in a low oil palm fruit set due to rain [51]. This is supported by Fatihah et al. [42]. Nevertheless, according to Rizuan et al. [48], the dry-season was more conducive for the EK population on the male inflorescence spikelet; no drop in pollinating efficiency was observed during the dry season. The Malaysia Department of Information on Climate (MDIC) [52] reported that the climate is equatorial in Malaysia, an Asian state situated north of the equator with humid weather year-round. It is difficult to find a region with rainfall less than 100 mm per month or 2000 mm per year; however, on average, April is considered as the wettest, with June as the driest month, and with average daily temperatures between 21 °C and 32 °C [52].

The application of pesticides such as *Cypermethrin* and *Chlorantraniliprole* in pest control in oil palm could affect the population abundance of *E. kamerunicus* [53]. Hybrids ECPHP550 followed by ECPHP500 recorded the highest weevil population. These two hybrids were developed from different genetic origins, but their male *pisifera* parents are good combiners with good reproductive features that may have attracted the EK. Soh et al. [6] reported that AVROS *pisiferas* are well known for their precocious bearing, vigorous growth, thick mesocarp, thin shell, and conferring characteristics of high oil yield. Similarly, Arolu et al. [54] reported that Nigeria *pisifera* is a good combiner with the attribute of dwarf characteristics for yield improvement.

The trial mean of the EK population abundance was 973.68 weevils, with the highest in hybrid ECPHP550. The EK population was low, especially the male *E. kamerunicus* when compared with other researchers’ findings. Rizuan et al. [48] reported that the actual population density of *E. kamerunicus* was 2883 weevils per palm. Meléndez and Ponce [55], reported that EK’s population density depends on the number of male inflorescences and the genetic origin of the palm species and its population. HA and Dzulhelmi [50] reported that the decline in fruit-set was likely due to an increase in natural enemies or insecticide applications against the *E. kamerunicus* or other predators. The yellow-vented predator bird, *Pycnonotus qoiavier*, was affirmed in Malaysian palm plantations to consume this noteworthy oil palm pollinator weevil as over 80% of its diet [49]. The variation in *E. kamerunicus* population among the hybrids may also be associated with the weevils being less active at pollination, especially during unfavorable conditions. Prasetyo et al. [29] reported that high rainfall has a major impact on the EK population and EK’s aggressiveness. Similarly, based on EK’s diminutive body structure, high wind velocity may be liable for the variation in EK population, especially in taller palms, because some hybrids’ palms were taller than others.

There was a significant positive weak relationship of SPL with the EK population on all the days (1–6) of anthesis. However, there was a perfect significant positive relationship of MEK/S with EKD2 and EKD3, although MEK/S had a moderate positive relationship with EKD1, EKD4, and EKD5, and a weak positive correlation with EKD6 was observed. This implies that the population density of the weevil starts on EKD1, increases on EKD2, and further increase on EKD3. Among the days of anthesis, EKD2 and EKD3 presented an increase in MEK/S. The EK population density started declining on EKD4 and EKD5, with fewer or no EK weevils found on EKD6. This result follows Yue et al. [41]. Additionally, PD/EK had a strong positive correlation with MEK/S. This observation exhibited that an increase in the mean number of EK per spikelet will enhance an increase in EK population density.

Similarly, the number of spikelets per male inflorescences at anthesis had a moderately positive significant correlation with PD/EK, indicating that spikelets with a strong scent/aroma will increase EK population density in palm plantations. Achieving a good fruit-set in oil palm primarily depended on the population and efficiency of *E. kamerunicus* which, in turn, relied on several components such as anthesising male inflorescence, number of spikelets, and the ability of the anthesising male inflorescence to produce a stronger aroma. According to Fatihah et al. [42], in determining the population density of EK in oil palm *dura* × *pisifera* hybrids, the number of male inflorescences and spikelets plays a major role. Therefore, hybrids with better male inflorescence production traits with a stronger aroma of the male flower could be a necessity in improving the population density of EK, especially the male EK for better pollination to achieve a better fruit-set.

Parental origin of T × N in producing the highest number of spikelets mean per male inflorescence (SM/MI), followed by A × AVROS, may be due to the vigor growth and yield attributes of their *pisifera* parents. Similarly, the spikelet length (SPL) among the genetic origins was not significant. However, the longest SPL was observed in A × AVROS, DJL × AVROS, DB × AVROS, and DUR × AVROS. This may be due to the use of the same male AVROS *pisifera* in their hybridization phases. The population density of EK by genetic origins had a parental mean of 963.24 weevils per palm. It was observed that 40% of the parental origins recorded higher PD/EK than the parental mean. Indicating that even among the parents from which the 24 hybrids used in this study were developed, there was a variation in their EK population density. A large population of the pollinator weevils occasionally could be advantageous, because it could result in lowering the performance of the oil palm fruit-set [48]. Conversely, Kouakou et al. [18] reported that the proportion of the oil palm bunch fruit-set is strongly related to the EK population density.

Deli Banting × AVROS recorded the best performance of genetic origins in terms of EK population density based on the current experimental findings, the highest population density of the pollinator weevil. From personal field observations, the outstanding performance of DB × AVROS in recording the highest EK population may be attributed to its stronger aroma produced by its anthesising male inflorescences, which may have attracted EK during pollination. Its tall nature coupled with its longer spikelets may have enhanced the weevil population density compared to the rest of the other parental origins used in the study. AVROS *psiferas* have been documented to be good combiners and are well-known worldwide for their active growth and high yield with thick mesocarp conferring attributes [6].

## 5. Conclusions

The present study assessed variation in the population density of *E. kamerunicus* in biparental D × P hybrids. Among the hybrids, the male weevil population was lower than the female. Significant variation was observed in anthesis days, with an increase in EK population from the first day of anthesis to the third day, while a population decline was recorded for the subsequent anthesis days. The variance in the number of weevils between hybrids significantly influenced the weevil population on the second and third days of anthesis. The highest mean number of EK per spikelet and population density per palm were recorded in January, while the lowest value was recorded in August and September. Hybrid ECPHP550 recorded EK’s highest mean per spikelet and population density per palm, followed by hybrid ECPHP500. Additionally, a significant positive relationship was observed between EK’s population density with MEK/S and a moderate correlation with the number of spikelets per male inflorescence. Among the ten parental origins, Deli Banting × AVROS had the highest population density followed by Tanzania × Nigeria. Therefore, hybrids ECPHP550 and parental Deli Banting × AVROS could be of necessity to increase the population of this weevil, due to the quality features they presented for recording higher *E. kamerunicus* population density. However, if volatile production traits of the male inflorescence were investigated concurrently with population density of *E. kamerunicus* among the hybrids, the results obtained from this study could have presented further details.

## Figures and Tables

**Table 1:** List of *dura* × *pisifera* (D × P) hybrids used in this study.

S/N	Crossing Materials	Pedigree No. ♂ Palm × ♀ Palm	Code
1	AVROS × Deli Banting	0.394/456 × 0.279/24	ECPHP415
2	Nigeria × Deli Ulu Remis	0.337/552 × 0.338/361	ECPHP500
3	AVROS × Deli Banting	0.394/234 × 0.279/24	ECPHP550
4	AVROS × Deli Johor Labis	0.394/234 × 0.281/44	ECPHP618
5	AVROS × Deli Ulu Remis	0.174/480 × 0.254/191	PK4118
6	AVROS × Angola	0.174/480 × 0.311/405	PK4465
7	Nigeria × Tanzania	0.337/1092 × 0.256/2058	PK4474
8	AVROS × Angola	0.394/24 × 0.311/405	PK4482
9	AVROS × Angola	0.174/247 × 0.312/99	PK4504
10	Avros × Angola	0.174/211 × 0.311/269	PK4505
11	Yangambi × Deli Ulu Remis	0.395/204 × 0.332/451	PK4529
12	AVROS × Deli Ulu Remis	0.394/24 × 0.332/100	PK4535
13	Nigeria, × Angola	0.337/1092 × 0.312/682	PK4539
14	Nigeria × Deli Ulu Remis	0.337/1092 × 0.332/218	PK4540
15	Yangambi × Deli Ulu Remis	0.395/204 × 0.332/45	PK4548
16	AVROS × Deli Ulu Remis	0.395/419 × 0.332/278	PK4550
17	AVROS × Tanzania	0.394/24 × 0.256/2313	PK4570
18	AVROS × Deli Ulu Remis	0.395/419 × 0.332/340	PK4591
19	Nigeria × Deli Ulu Remis	0.337/554 × 0.332/220	PK4621
20	Nigeria × Deli Ulu Remis	0.337/1091 × 0.332/116	PK4648
21	Nigeria × Tanzania	0.337/1092 × 0.256/2425	PK4651
22	AVROS × Deli Ulu Remis	0.395/372 × 0.332/116	PK4674
23	Nigeria × Angola	0.337/291 × 0.312/1241	PK4679
24	Cameroon × Deli Serdang	0.219/1371 × 0.212/6	PK4841

Notes: AVROS, Algemene Vereniging; ECPHP, *Elaeis guineensis* crossing program Hulu Paka; PK, Porim Kluang; P, *pisifera* palm; D, *dura* palm.

**Table 2:** ANOVA and variance components for the population of *Elaeidobius kamerunicus* per spikelet on each day of anthesis.

S/V	DF	MD1	FD1	EKD1	MD2	FD2	EKD2	MD3	FD3	EKD3
Replications (R)	3	˂0.01 ^ns^	0.01 ^ns^	0.01 ^ns^	0.02 ^ns^	0.10 ^ns^	0.11 ^ns^	0.13 ^ns^	2.16 ^ns^	1.98 ^ns^
Hybrids (G)	23	0.01 ^ns^	0.08 **	0.10 **	0.11 **	1.29 **	1.87 **	0.38 **	5.92 **	8.87 **
Error (e)	60	˂0.01	0.01	0.02	0.02	12.17	0.28	4.33	0.76	1.10
Variance component										
σ^2^_g_		˂0.01 (13.33) ^+^	˂0.01 (55.24)	0.03 (60.00)	0.02 (50.00)	0.28 (58.33)	0.42 (60.87)	0.08 (53.33)	1.33 (63.64)	2.03 (65.48)
σ^2^_e_		˂0.01 (86.67)	˂0.01 (44.76)	0.02 (40.00)	0.02 (50.00)	0.2 (41.66)	0.27 (39.13)	0.07 (46.67)	0.76 (36.36)	1.07 (34.52)
σ^2^_ph_		˂0.01	˂0.01	0.05	0.05	0.48	0.69	0.15	2.09	3.10
Mean		0.22	1.08	1.3	1.73	6.27	7.99	3.42	12.94	16.36
Std Error		0.01	0.02	0.02	0.02	0.07	0.09	0.04	0.16	0.19
S/V	DF	MD4	FD4	EKD4	MD5	FD5	EKD5	MD6	FD6	EKD6
Replications (R)	3	0.10 ^ns^	1.57 *	1.75 *	0.01 ^ns^	0.18 ^ns^	0.22 ^ns^	˂0.01 ^ns^	˂0.01 ^ns^	˂0.01 ^ns^
Hybrids (G)	23	0.13 **	1.48 **	2.27 **	0.04 ^ns^	0.31 **	0.52 **	˂0.01 ^ns^	0.01 **	0.01 **
Error (e)	60	0.05	0.37	0.59	0.03	0.10	0.17	˂0.01	˂0.01	˂0.01
Variance component										
σ^2^_g_		0.02 (28.57)	0.30 (44.78)	0.46 (43.81)	˂0.01 (11.77)	0.06 (35.29)	0.10 (37.04)	˂0.01 (7.50)	˂0.01 (40.00)	˂0.01 (47.50)
σ^2^_e_		0.05 (71.43)	0.37 (55.22)	0.59 (56.19)	0.03 (88.23)	0.11 (64.71)	0.17 (62.96)	˂0.01 (92.50)	˂0.01 (60.00)	˂0.01 (52.50)
σ^2^_ph_		0.07	0.67	1.05	0.034	0.17	0.27	˂0.01	˂0.01	˂0.01
Mean		2.14	7.02	9.16	1.02	3.13	4.15	0.02	0.07	0.09
Stderr		0.03	0.09	0.11	0.02	0.04	0.06	˂0.01	0.01	0.01

Notes: S/V, source of variation; DF, degree of freedom; EK, *Elaeidobius kamerunicus*; MD1, male day-one; FD1, female day-one; EKD1, EK day-one; MD2, male day-two; FD2, female day-two; EKD2, EK day-two; MD3, male day-three; FD3, female day-three; EKD3, EK day-three; MD4, male day-four; FD4, female day-four; EKD4, EK day-four; MD5, male day-five; FD5, female day-five; EKD5, EK day-five; MD6, male day-six; FD6, female day-six, EKD6, EK day-six; ^ns^, non-significant at *p* > 0.05; * significant at *p* < 0.05; ** highly significant at *p* < 0.01; ()^+^, phenotypic variance in percentage; σ^2^_g_, variance in number of weevils between palm genotypes; σ^2^_e_, error variance; σ^2^_ph_, phenotypic variance; Stderr, standard error.

**Table 3:** Analysis of variance and monthly population mean ± standard error per spikelet and population density of *E. kamerunicus* in D × P hybrids.

S/V	DF	MEK/S	PD/EK
Replications (R)	3	3.41 ^ns^	203,442.10 ^ns^
Months (M)	11	684.25 **	18,934,909.40 **
Hybrids (G)	23	27.07 **	863,523.40 **
M*G	253	3.63 **	140,595.90 *
Error (e)	753	1.91	117,313.40
Mean		8.55	973.68
Stderr		0.10	18.31
No.	Month		
1	February 2019	10.14 ^c^ ± 0.15	1158.57 ^d^ ± 41.28
2	March 2019	11.41 ^b^ ± 0.27	1526.78 ^b^ ± 69.00
3	April 2019	7.57 ^d^ ± 0.14	709.34 ^ef^ ± 24.45
4	May 2019	5.92 ^e^ ± 0.22	548.02 ^f^ ± 31.91
5	June 2019	7.70 ^d^ ± 0.17	1216.00 ^cd^ ± 46.79
6	July 2019	8.22 ^d^ ± 0.17	653.38 ^ef^ ± 25.38
7	August 2019	3.96 ^f^ ± 0.12	360.63 ^g^ ± 14.24
8	September 2019	4.36 ^f^ ± 0.17	359.57 ^g^ ± 16.71
9	October 2019	10.62 ^c^ ± 0.16	1218.66 ^cd^ ±34.92
10	November 2019	8.10 ^d^ ±0.16	729.16 ^e^ ± 23.24
11	December 2019	11.79 ^b^ ±0.18	1357.58 ^bc^ ± 49.55
12	January 2020	12.81 ^a^ ± 0.23	1846.49 ^a^ ± 60.69
Hybrid			
1	ECPHP415	8.91 ^b–e^ ± 0.13	1104.63 ^a–e^ ± 99.34
2	ECPHP500	9.80 ^ab^ ± 0.26	1226.82 ^ab^ ± 61.21
3	ECPHP550	10.25 ^a^ ± 0.11	1241.39 ^a^ ± 73.74
4	ECPHP618	8.74 ^b–e^ ± 0.32	959.91 ^b–f^ ± 72.82
5	PK4118	9.01 ^b–e^ ± 0.22	1149.72 ^a–c^ ± 62.19
6	PK4465	7.99 ^d–f^ ± 0.05	975.24 ^a–f^ ± 40.75
7	PK4474	8.49 ^c–f^ ± 0.2	1053.40 ^a–f^ ± 6.06
8	PK4482	8.06 ^d–f^ ± 0.21	941.35 ^c–f^ ± 74.68
9	PK4504	8.67 ^c–e^ ± 0.5	980.68 ^a–f^ ± 60.58
10	PK4505	8.77 ^b–e^ ± 0.19	988.46 ^a–f^ ± 27.02
11	PK4529	7.94 ^d–f^ ± 0.38	840.10 ^d–g^ ± 37.38
12	PK4535	8.47 ^c–f^ ± 0.19	899.19 ^c–g^ ± 49.06
13	PK4539	7.42 ^fg^ ± 0.14	813.47 ^fg^ ± 24.54
14	PK4540	7.91 ^ef^ ± 0.15	880.07 ^c–g^ ± 19.5
15	PK4548	8.25 ^d–f^ ± 0.39	833.60 ^e–g^ ± 22.37
16	PK4550	8.38 ^c–f^ ± 0.21	986.65 ^a–f^ ± 58.97
17	PK4570	8.33 ^c–f^ ± 0.16	885.42 ^c–g^ ± 30.67
18	PK4591	8.51 ^c–f^ ± 0.23	928.37 ^c–f^ ± 39.54
19	PK4621	6.49 ^g^ ± 0.33	629.90 ^g^ ± 44.09
20	PK4648	9.43 ^a–c^ ± 0.2	1108.05 ^a–d^ ± 94.8
21	PK4651	8.96 ^b–e^ ± 0.26	1023.26 ^a–f^ ± 46.12
22	PK4674	8.33 ^c–f^ ± 0.41	992.81 ^a–f^ ± 47.68
23	PK4679	9.04 ^b–d^ ± 0.28	977.63 ^a–f^ ± 28.8
24	PK4841	8.53 ^c–e^ ± 0.27	919.31 ^c–f^ ± 66.73
Mean ± Stderr		8.55 ± 0.10	973.68 ± 18.31

**Notes:** S/V, source of variation; DF, degree of freedom; MEK/S, mean of *Elaeidobius kamerunicus* spikelet; PD/EK, population density of *Elaeidobius kamerunicus*; ECPHP, *Elaeis guineensis* crossing program Hulu Paka; PK, Porim Kluang; Stderr, standard error; ^ns^, non-significant at *p* > 0.05; * significant at *p* < 0.05; ** highly significant at *p* < 0.01. Means with the same letter of alphabet in the same column are not significantly different at *p* < 0.05 with Tukey’s studentized range (HSD).

## Data Availability

The datasets used and analyzed during the current study are available within the manuscript.
